# Strong association between metabolically-abnormal obesity and gallstone disease in adults under 50 years

**DOI:** 10.1186/s12876-019-1032-y

**Published:** 2019-07-04

**Authors:** Pei-yuan Su, Yu-Chun Hsu, Yu-fang Cheng, Chew-Teng Kor, Wei-Wen Su

**Affiliations:** 1 Department of Gastroenterology, Changhua Christian Hospital, Changhua, Taiwan; 2 Division of Endocrinology and Metabolism, Changhua Christian Hospital, Changhua, Taiwan; 3 Internal Medicine Research Center, Changhua Christian Hospital, Changhua, Taiwan

**Keywords:** Gallstones, Risk factors, Metabolic syndrome, Obesity

## Abstract

**Background:**

Age, obesity, and metabolic syndrome are known risk factors for gallstones; however, the combined impact of these different risk factors on gallstone formation has not yet been examined.

**Methods:**

This retrospective, cross-sectional study involved 3190 participants, including 207 participants (6.5%) with gallstones and 986 (30.9%) with metabolic syndrome. Participants were divided into four phenotypes according to metabolic syndrome and obesity status: 1378 participants were metabolically healthy and non-obese (MHNO); 826 were metabolically healthy but obese (MHO); 185 were metabolically abnormal but not obese (MANO); and 801 participants were metabolically abnormal and obese (MAO).

**Results:**

The MAO and MANO phenotypes had more gallstones than the MHO and MHNO phenotypes, regardless of age (< 50 or ≥ 50 years old). Multivariate analyses showed that phenotype was an independent risk factor for gallstones in participants < 50 years old (odds ratio (OR) = 1.73, 95% confidence interval (CI) = 1.32–2.28). Younger participants also had a higher risk of gallstones in the MAO (OR = 5.41, 95% CI = 2.31–12.66), MANO (OR = 3.18, 95% CI = 0.86–11.75), and MHO (OR = 2.17, 95% CI = 0.90–5.22) phenotypes than the MHNO phenotype.

**Conclusions:**

Our retrospective results demonstrate an increased association of gallstones in younger people (< 50 years old) with metabolic syndrome and obesity.

**Electronic supplementary material:**

The online version of this article (10.1186/s12876-019-1032-y) contains supplementary material, which is available to authorized users.

## Background

Gallstones and their associated complications are common conditions worldwide. Approximately 6–20% of the population has gallstones and some studies have indicated rates of screen-detected gallstones between 0.6 and 1.39% per year in Taiwan and Europe [1–3]. Moreover, specific groups, such as those with cirrhosis and Type 2 diabetes mellitus (DM), have gallstone incidence rates of 2–5% per year [4]. More than 20% of people with gallstones develop biliary colic or gallstone-associated complications, including acute pancreatitis, cholangitis, cholecystitis, and common bile duct stones [5].

Several studies have shown that age, sex, body mass index (BMI), non-alcoholic fatty liver, Type 2 DM, and insulin resistance are associated with gallstones [6–8]. Furthermore, people with greater incidence of metabolic abnormality have a higher risk of developing gallstones [9]. BMI and obesity also increase risk of gallstone development [10, 11]. Because obese individuals with different metabolic profiles can present with distinct cardiovascular or diabetes outcomes, many investigators have developed obesity sub-types, using metabolic factors, as four phenotypes: metabolically-healthy and non-obese (MHNO), metabolically-healthy but obese (MHO), metabolically-abnormal but not obese (MANO) and metabolically-abnormal and obese (MAO) [12, 13]. The studies found that the MAO phenotype has the highest risk of cardiovascular disease, chronic kidney disease (CKD), and advanced colorectal neoplasia compared to MHO or MANO individuals [14–16]. However, the association between gallstones and these four phenotypes is still unknown. Age is also a major risk factor that is positively correlated with gallstone development. However, it is not known whether these risk factors contribute equally in younger and older populations. Our study aimed to analyze the impact of risk factors including age, metabolic syndrome, and obesity on gallstone formation.

## Methods

This retrospective, cross-sectional study included patients who received voluntary physical check-ups from the Health Examination Center of Changhua Christian Hospital, Changhua City, Taiwan between 2010 and 2014. A total of 4456 participants were initially enrolled in this study; however, 774 were excluded because:

- 51 had incomplete data to define their metabolic syndrome;
- 415 underwent repeat examination during the study period;
- 257 refused to undergo abdominal ultrasound;
- 491 had incomplete data for hepatitis B or C; and
- 51 had already undergone cholecystectomy.

In the end, 3190 participants were eligible for further analysis. All participants in the final group received blood tests (including glucose, glycated hemoglobin (HbA1c), cholesterol, high-density lipoprotein cholesterol (HDL-C), low-density lipoprotein cholesterol (LDL-C), triglycerides, hepatitis B surface antigen, and anti-hepatitis C virus antibody), blood pressure measurement, waist circumference measurements, and abdominal sonography. The Ethics Committee of Changhua Christian Hospital approved the study protocol used in this work (CCH IRB No: 150807).

A gallstone was defined as a hyperechoic area with a typical acoustic shadow and positional change by sonography and was diagnosed by an experienced gastroenterologist who has more than 5-years of experience and was unaware of personal history or laboratory data. Cholecystectomy was defined as an invisible gallbladder can be identified by sonography or by a personal history of cholecystectomy. Metabolic syndrome was defined as fulfilling at least three of the five Adult Treatment Panel III criteria: (1) central obesity, defined as a waist circumference of ≥90 cm in Asian men and ≥ 80 cm in Asian women; (2) low HDL-C levels, defined as fasting HDL-C < 40 mg/dL in men and < 50 mg/dL in women; (3) hypertension, defined as systolic pressure ≥ 130 mmHg and/or diastolic ≥85 mmHg, or in patients with a history of hypertension receiving antihypertensive therapy; (4) high triglyceridemia, defined as fasting triglyceride levels ≥150 mg/dL; and (5) hyperglycemia, defined as fasting glucose level ≥ 100 mg/dL, or in patients with a history of diabetes receiving medications to control elevated glucose [17]. Obesity was defined as BMI ≥ 25 kg/cm^2^ in the study by using the classification for adult Asians proposed by WHO in 2000 [18].

Participants were further divided into four phenotypes according to metabolic syndrome and obesity profile: MHNO included participants without both metabolic syndrome and obesity; MHO included those without metabolic syndrome but with obesity; MANO included participants with metabolic syndrome but without obesity; and MAO included those who had metabolic syndrome combined with obesity.

### Statistical analyses

Continuous variables are presented as mean ± standard deviation and were compared using an ANOVA test. Categorical data were compared using a chi-square test. Single-variable, binary logistic regression and non-parsimonious multivariable logistic regression analysis were used to analyze the significance of variables in gallstones. Statistical analyses were performed using PASW Statistics version 18 (formerly SPSS; IBM, Hong Kong). *P* <  0.05 (two-tailed) was considered statistically significant.

## Results

### Baseline characteristics

There were 3190 participants enrolled for the final analysis, including 207 participants (6.5%) with gallstones and 986 (30.9%) with metabolic syndrome. The numbers and rates of participants in each phenotype were 1633 (51.2%) for the MHNO phenotype, 571 (17.9%) for the MHO phenotype, 292 (9.2%) for the MANO phenotype, and 694 (21.8%) for the MAO phenotype. Participants in the MAO and the MANO phenotypes were older and had higher blood pressure and more abnormal levels of blood glucose, liver function tests (aspartate transaminase, alanine transaminase, alkaline phosphatase, and gamma-glutamyl transferase, except bilirubin), lipid profiles, uric acid, and renal function (blood urea nitrogen and creatinine) compared to those in the MHNO and MHO phenotypes. Detailed results are listed in Table 1 (Additional file 1: Table S1).

**Table 1:** Baseline characteristics of participants according to metabolic syndrome and obesity profile

	MHNO (*n* = 1633)	MHO (*n* = 571)	MANO (*n* = 292)	MAO (*n* = 694)	*p* value
Age, years	48.4 ± 12.5	50 ± 12.2	55.9 ± 10.8	53.3 ± 11.8	< 0.001
Sex,Male (%)	49.6%	71.6%	58.9%	67.4%	< 0.001
Waist circumference (cm)	75.8 ± 6.8	87.5 ± 6.9	82.5 ± 5.8	92.5 ± 7.5	< 0.001
SBP (mmHg)	120 ± 17	125 ± 16	133 ± 18	134 ± 16	< 0.001
DBP (mmHg)	77 ± 10	80 ± 10	83 ± 9	85 ± 10	< 0.001
BMI (kg/m^2^)	21.8 ± 2.0	26.9 ± 2.0	23.3 ± 1.3	28.3 ± 2.6	< 0.001
FBG (mg/dL)	94 ± 19	95 ± 13	114 ± 40	112 ± 32	< 0.001
HbA1C	5.5 ± 0.8	5.6 ± 0.5	6.2 ± 1.4	6.1 ± 1.2	< 0.001
Bil-T	0.97 ± 0.42	1.00 ± 0.44	0.95 ± 0.39	0.98 ± 0.42	0.207
GOT	25 ± 11	28 ± 13	29 ± 12	31 ± 16	< 0.001
GPT	24 ± 16	32 ± 20	33 ± 20	37 ± 24	< 0.001
r-GT	23 ± 30	29 ± 28	35 ± 32	41 ± 57	< 0.001
ALP	58 ± 18	58 ± 15	65 ± 18	64 ± 19	< 0.001
Total cholesterol (TC)	197 ± 36	203 ± 37	201 ± 44	201 ± 39	0.002
HDL (mg/dL)	54 ± 14	48 ± 10	41 ± 9	40 ± 9	< 0.001
LDL (mg/dL)	122 ± 32	132 ± 33	123 ± 36	126 ± 33	< 0.001
Triglyceride (mg/dL)	88 ± 49	110 ± 75	189 ± 111	188 ± 133	< 0.001
Uric acid	5.4 ± 1.4	6.3 ± 1.4	6.1 ± 1.5	6.7 ± 1.5	< 0.001
BUN	9.6 ± 3.8	10.3 ± 3.5	10.8 ± 4.8	10.8 ± 4.3	< 0.001
Cr	0.78 ± 0.31	0.85 ± 0.21	0.82 ± 0.31	0.87 ± 0.30	< 0.001
HBV	11.5%	12.4%	7.9%	8.5%	0.033
HCV	3.4%	3.0%	6.2%	3.3%	0.084
GB stones	4.4%	6.5%	12.3%	8.9%	< 0.001

*ALP* alkaline phosphatase, *ALT* alanine transaminase, *AST* aspartate transaminase, *Bil-T* total bilirubin, *BMI* body mass index, *BUN* blood urea nitrogen, *Cr* creatinine, *DBP* diastolic blood pressure, *FBG* fasting blood glucose, *HbA1c* glycated hemoglobin, *HBV* hepatitis B virus, *HCV* hepatitis C virus, *HDL* high-density lipoprotein, *LDL* low-density lipoprotein, *MANO* metabolically abnormal but not obese, *MAO* metabolically abnormal and obese, *MHNO* metabolically healthy and non-obese, *MHO* metabolically healthy but obese, *r-GT* gamma-glutamyl transferase, *SBP* systolic blood pressure

A total of 98 (10%) participants with metabolic syndrome had gallstones but only 109 (5%) had gallstones among those without metabolic syndrome. Age, HCV infection and four phenotypes were the risk factors for gallstones after non-parsimonious multivariable logistic regression (Additional file 2: Table S2). The median age in the study was 50 years old and the interaction effect is statistically significant in subgroups divided by age below and above 50. Table 2 shows higher rates of gallstones in both the MAO and MANO phenotypes compared to the MHO and MHNO phenotypes, regardless of age (< 50 or ≥ 50 years old). The rate of gallstones was similar in younger participants (< 50 years old) in the MAO phenotype compared to older participants (≥50 years old) in the MHNO phenotype (7.2% vs. 6.8%; *P* = 0.837).

**Table 2:** Gallstone rates of the 4 phenotypes according to metabolic syndrome and obesity profile in participants < 50 and ≥ 50 years

Age	Phenotypes	Non-GB stone (*n* = 2983)		GB stone (*n* = 207)		*p* value
		n	%	n	%	
Age < 50	MHNO (*n* = 859)	840	97.8%	19	2.2%	0.002
	MHO (*n* = 265)	255	96.2%	10	3.8%	
	MANO (*n* = 71)	68	95.8%	3	4.2%	
	MAO (*n* = 249)	231	92.8%	18	7.2%	
Age ≥ 50	MHNO (*n* = 774)	721	93.2%	53	6.8%	0.002
	MHO (*n* = 306)	279	91.2%	27	8.8%	
	MANO (*n* = 221)	188	85.1%	33	14.9%	
	MAO (*n* = 445)	401	90.1%	44	9.9%	

*MANO* metabolically abnormal but not obese, *MAO* metabolically abnormal and obese, *MHNO* metabolically healthy and non-obese, *MHO* metabolically healthy but obese

As shown in Table 3, we used univariate and multivariate regression analysis to determine the risk factors for gallstones in two the different groups divided by age. The results revealed that the four phenotypes— according to metabolic syndrome and obesity profile— were the only independent risk factors for gallstones in participants younger than 50 years old (odds ratio (OR) = 1.73, 95% confidence interval (CI) = 1.32–2.28; *P* <  0.001). However, in participants older than 50, only HCV infection was a significant independent factor for gallstones (OR = 2.58, 95% CI = 1.43–4.68; *P* <  0.05). Subgroup analyses were performed after multivariate regression to identify effects in each group of participants. The results are listed in Table 4. When MHNO was used as a reference, the ORs in each phenotype of participants (< 50 years old) were MHO (OR = 2.17, 95% CI = 0.86–11.75; *P* = 0.08), MANO (OR = 3.18, 95% CI = 0.86–11.75; *P* = 0.08) and MAO (OR = 5.41, 95% CI = 2.31–12.66; *P* <  0.001). No statistically significant differences were found between MHNO, MHO and MAO phenotypes (≥50 years old).

**Table 3:** Risk of gallstones in multivariate analyses of two different age groups

Variables	Age < 50					Age ≥ 50				
	cOR	95% CI	adj. OR	95% CI	p	cOR	95% CI	adj. OR	95% CI	p
Sex	0.90	0.51–1.59	0.92	0.32–2.60	0.87	1.05	0.75–1.47	0.79	0.49–1.27	0.32
Bil-T	0.74	0.36–1.51	1.08	0.49–2.38	0.85	1.46	1.01–2.11	1.44	0.95–2.16	0.08
GOT	0.97	0.94–1.01	0.94	0.87–1.02	0.13	1.02	1.01–1.03	1.01	0.99–1.03	0.38
GPT	1.00	0.98–1.01	1.02	0.98–1.05	0.31	1.01	1.00–1.02	1.00	0.99–1.02	0.88
rGT	1.00	0.98–1.01	1.00	0.98–1.02	0.89	1.00	1.00–1.01	1.00	1.00–1.01	0.28
ALP	0.99	0.97–1.01	0.99	0.97–1.01	0.45	1.01	1.00–1.01	1.00	1.00–1.01	0.34
TC	0.99	0.98–1.00	0.98	0.96–1.01	0.15	1.00	0.99–1.00	0.99	0.98–1.01	0.30
LDL	1.00	0.99–1.01	1.01	0.99–1.04	0.38	1.00	0.99–1.00	1.01	0.99–1.02	0.43
Uric acid	1.01	0.84–1.21	0.94	0.71–1.25	0.65	1.18	1.06–1.31	1.13	0.98–1.30	0.09
BUN	1.02	0.95–1.10	1.06	0.95–1.18	0.32	1.02	0.98–1.05	1.00	0.95–1.05	0.98
Cr	0.57	0.11–2.86	0.50	0.04–6.37	0.59	1.34	0.81–2.20	1.23	0.55–2.78	0.61
HBV	1.22	0.54–2.76	1.14	0.46–2.80	0.77	0.91	0.51–1.61	0.99	0.54–1.80	0.97
HCV	–	–	0.00	< 0.001	1.00	2.86	1.68–4.88	2.58	1.43–4.68	< 0.05
Four phenotypes^a^	1.50	1.21–1.86	1.73	1.32–2.28	< 0.001	1.18	1.04–1.34	1.10	0.94–1.27	0.23

*ALP* alkaline phosphatase, *ALT* alanine transaminase, *AST* aspartate transaminase, *Bil-T* total bilirubin, *ALP* alkaline phosphatase, *AST* aspartate transaminase, *Bil-T* total bilirubin, *LDL* low-density lipoprotein, *TC* total cholesterol, *HBV* hepatitis B virus, *HCV* hepatitis C virus; ^a^Four phenotypes: MHNO, MHO, MANO and MAO

**Table 4:** Gallstones in the four different phenotypes by metabolic syndrome and obesity profile according to age (< 50 and ≥ 50 years)

Age (years)	Phenotypes	OR	95% CI	p
Age < 50	MHNO	1 Reference		
	MHO	2.17	0.90–5.22	0.08
	MANO	3.18	0.86–11.75	0.08
	MAO	5.41	2.31–12.66	< 0.001
Age ≥ 50	MHNO	1 Reference		
	MHO	1.28	0.77–2.15	0.34
	MANO	1.80	1.06–3.05	0.03
	MAO	1.24	0.77–1.99	0.38

*MAO* metabolically abnormal and obese, *MHNO* metabolically healthy and non-obese, *MHO* metabolically healthy but obese, *MANO* metabolically abnormal but not obese

## Discussion

Many studies have shown that several independent factors are associated with gallstone development, including age, sex, metabolic syndromes, obesity, Type 2 DM, BMI, HCV, and CKD [2, 5, 19]. While age is a very strong risk factor for gallstones, it is unknown whether these risk factors contribute differently toward gallstone formation in younger people. This study is the first to analyze these risk factors for gallstones according to different age groups in an Asian population. Our results showed that the combination of metabolic syndrome and obesity posed the greatest likelihood of gallstone formation in people younger than 50 years old. However, in people older than 50, HCV was the major associated factor for gallstone formation.

Metabolic syndrome and obesity are two of the main risk factors for gallstones and the prevalence of gallstones is higher in individuals with a greater number of abnormal metabolic factors [3, 6, 9, 20]. Some studies have shown waist circumference, HDL, and blood glucose as major factors for gallstone formation [3, 20]. The same was also noted for obesity; high BMI was associated with prevalence of gallstones [10, 11]. However, it is unclear whether both metabolic syndrome and obesity, or only one of these factors, contributes to the high prevalence of gallstones. Many studies have attempted to classify four different phenotypes—MAO, MANO, MHO, and MHNO—according to obesity and metabolic health status [21–24]. Risk of diabetes, cardiovascular disease, and CKD remained inconclusive in the MHO phenotype. Hashimoto et al. showed that the MHO phenotype had a similar risk of CKD compared with the MHNO phenotype [16]. Luo et al. showed no increased risk of diabetes or cardiovascular disease in the MHO phenotype. Moreover, there was no significant difference in the risk of advanced colorectal neoplasia between the MHO and MHNO but MANO and MAO are increased risk of metachronous colorectal neoplasia in a study by Kim et al. [14] In contrast, Ryoo et al. concluded that the MHO phenotype had an increased risk of diabetes according to the degree of obesity [25]. In addition to age, the levels of serum uric acid, liver function tests, and renal function significantly differed amongst the four groups, being more abnormal in MAO and MANO than MHO and MHNO in our study. However, after adjusting all these factors by multivariate regression, only age, HCV infection and the four phenotypes were independent factors associated with gallstones. We also found that the MAO phenotype had a higher prevalence (7.2%) and odds ratio (5.41) for gallstones than the other three phenotypes (prevalence 2.2–4.8%; OR: 2.17–3.78) in people younger than 50.

Many studies have reported age as a major risk factor for gallstones [8, 26–28]. In addition, the elderly population has been shown to have a higher rate of gallstone-associated complications, such as cholecystitis and gallstone pancreatitis, and a higher rate of surgical complications after cholecystectomy [29, 30]. A study by Li et al. revealed HCV to be a substantial risk factor for gallstones in populations older than 60 (adjusted OR: 2.394 (1.066–5.375)) [31]. We found a similar result in our study: in the older groups (≥50 years old), HCV infection was the only significant risk factor for gallstones. However, this effect was not seen in the younger group (< 50 years old). The prevalence of gallstones in younger participants (< 50 years old) with both metabolic syndrome and obesity was similar to that observed in metabolically healthy and non-obese older participants (≥50 years old) (7.2% vs. 6.8%; *P* = 0.837). Therefore, the effect of metabolic syndrome and obesity on gallstones in younger participants was similar to the effect of age on gallstones in older participants without metabolic syndrome and obesity. The HCV infection rate was lower in the younger population, with the prevalence of both metabolic syndrome and obesity in young adults increasing gradually in Taiwan. HCV may also have a close association with insulin resistance and hepatic steatosis [32, 33]. All these factors (HCV infection, insulin resistance, or hepatic steatosis) were positively correlated with gallstones [31, 34]. Whether the relationship between HCV and metabolic syndrome has a synergistic effect or a causal relationship on gallstone formation requires further study [35]. Our study also showed that the effect of metabolic syndrome and obesity on gallstones was stronger in younger participants. The effect would be decreased in older participants because they had more interactive effects with factors that were also associated with gallstones, such as Type 2 DM, HCV, and CKD.

Although more than 3000 participants were included in the study, there were still several limitations. Firstly, we did not distinguish between the types of gallstones included (cholesterol or pigment stones) because we used sonographic findings. Secondly, this was a cross-sectional, retrospective study; the incident rate of gallstones in different age groups with metabolic syndrome and obesity needs to be confirmed using longitudinal studies. Thirdly, the study lacked some published data that is currently available in the literature regarding other risk factors of gallstones, such as smoking, drinking alcohol, exercise and lifestyle, pregnancy, oral contraceptive use, or family history. Fourth, the study groups were from an Asian population in Taiwan; the incidences of viral hepatitis B or C infection and definitions of obesity differ between the Eastern and Western worlds. Further studies are needed using data from different races for comparison with the current results.

## Conclusions

In conclusion, the present study has shown an increased association of gallstones in younger people with metabolic syndrome and obesity. The prevalence of gallstones in younger people with metabolic abnormality and obesity was equal to that of metabolically-healthy and non-obese elderly people. Diet and exercise intervention may be beneficial for high-risk groups to prevent gallstone formation; however, additional studies are required to confirm these findings.

## Additional files

Additional file 1: **Table S1.** Baseline characteristics of participants according to participants < 50 and ≥ 50 years. (DOCX 16 kb)

Additional file 2: **Table S2.** Risk of gallstones in multivariate analyses. (DOCX 16 kb)

## Data Availability

The datasets used and analysed during the current study are available from the corresponding author on reasonable request.

## Abbreviations

ALP: Alkaline phosphatase
ALT: Alanine transaminase
AST: Aspartate transaminase
BMI: Body mass index
BUN: Blood urea nitrogen
CKD: Chronic kidney disease
DBP: Diastolic blood pressure
DM: Diabetes mellitus
FBG: Fasting blood glucose
HBV: Hepatitis B virus
HCV: Hepatitis C virus
HDL: High-density lipoprotein
LDL: Low-density lipoprotein
MANO: Metabolically-abnormal but not obese
MAO: Metabolically-abnormal and obese
MHNO: Metabolically-healthy and non-obese
MHO: Metabolically-healthy but obese
OR: Odds ratio
SBP: Systolic blood pressure
