# Supplementary material for: Strong association between metabolically-abnormal obesity and gallstone disease in adults under 50 years
Source: BMC Gastroenterol. 2019 Jul 4;19:117. doi: 10.1186/s12876-019-1032-y (PMC6610843; doi:10.1186/s12876-019-1032-y)
Supplement: Supplementary file 1 — Table S1. Baseline characteristics of participants according to participants < 50 and ≥ 50 years. (DOCX 16 kb) [file 12876_2019_1032_MOESM1_ESM.docx]

Additional file 1: Table S1. Baseline characteristics of participants according to participants <50 and ≥50 years

|  | Age < 50 | Age ≥ 50 | p value |
| --- | --- | --- | --- |
| Age, years | 39.2 ± 6.9 | 59.7 ± 7.2 | <0.001 |
| Sex, Male (%) | 58.45% | 58.13% | 0.857 |
| Waist circumference (cm) | 80.4 ± 10.3 | 83.6 ± 9.2 | <0.001 |
| SBP (mmHg) | 119 ± 15 | 131 ± 18 | <0.001 |
| DBP (mmHg) | 79 ± 10 | 81 ± 10 | <0.001 |
| BMI (kg/m^2^) | 23.9 ± 3.7 | 24.6 ± 3.3 | <0.001 |
| FBG (mg/dL) | 94 ± 19 | 105 ± 29 | <0.001 |
| HbA1C | 5.5 ± 0.8 | 5.9 ± 1 | <0.001 |
| Bil-T | 0.98 ± 0.45 | 0.97 ± 0.39 | 0.666 |
| GOT | 26 ± 12 | 29 ± 14 | <0.001 |
| GPT | 29 ± 23 | 29 ± 18 | 0.628 |
| r-GT | 29 ± 43 | 30 ± 34 | 0.409 |
| ALP | 56 ± 15 | 64 ± 19 | <0.001 |
| Total cholesterol (TC) | 195 ± 37 | 203 ± 38 | <0.001 |
| HDL (mg/dL) | 49 ± 14 | 49 ± 13 | 0.837 |
| LDL (mg/dL) | 121 ± 32 | 127 ± 33 | <0.001 |
| Triglyceride (mg/dL) | 122.4 ± 110.45 | 123.75 ± 81.82 | 0.701 |
| Uric acid | 5.8 ± 1.5 | 6 ± 1.5 | <0.001 |
| BUN | 8.9 ± 3.1 | 11.1 ± 4.3 | <0.001 |
| Cr | 0.79 ± 0.3 | 0.83 ± 0.29 | <0.001 |
| HBV | 11.84% | 9.68% | 0.049 |
| HCV | 1.45% | 5.27% | <0.001 |
| MHNO | 59.49% | 44.33% | <0.001 |
| MHO | 18.35% | 17.53% | 0.576 |
| MANO | 4.92% | 12.66% | <0.001 |
| MAO | 17.24% | 25.49% | <0.001 |
| GB stone | 3.50% | 9.00% | <0.001 |

ALP, alkaline phosphatase; ALT, alanine transaminase; AST, aspartate transaminase; Bil-T, total bilirubin; BMI, body mass index; BUN, blood urea nitrogen; Cr, creatinine; DBP, diastolic blood pressure; FBG, fasting blood glucose; HbA1c, glycated hemoglobin; HBV, hepatitis B virus; HCV, hepatitis C virus; HDL, high-density lipoprotein; LDL, low-density lipoprotein; MANO, metabolically abnormal but not obese; MAO, metabolically abnormal and obese; MHNO, metabolically healthy and non-obese; MHO, metabolically healthy but obese; r-GT gamma-glutamyl transferase; SBP, systolic blood pressure.
