# Supplementary material for: Strong association between metabolically-abnormal obesity and gallstone disease in adults under 50 years
Source: BMC Gastroenterol. 2019 Jul 4;19:117. doi: 10.1186/s12876-019-1032-y (PMC6610843; doi:10.1186/s12876-019-1032-y)
Supplement: Supplementary file 2 — Table S2. Risk of gallstones in multivariate analyses. (DOCX 16 kb) [file 12876_2019_1032_MOESM2_ESM.docx]

Additional file 2: Table S2. Risk of gallstones in multivariate analyses

| Variables | cOR | 95% CI | p |  | adj.OR | 95% CI | p |
| --- | --- | --- | --- | --- | --- | --- | --- |
| Age | 1.05 | 1.04-1.06 | < 0.001 |  | 1.05 | 1.03-1.06 | < 0.001 |
| Sex | 1.01 | 0.76-1.34 | 0.957 |  | 0.80 | 0.53-1.19 | 0.272 |
| Bil_T | 1.20 | 0.88-1.65 | 0.249 |  | 1.26 | 0.88-1.80 | 0.202 |
| GOT | 1.01 | 1.01-1.02 | < 0.001 |  | 1.00 | 0.99-1.02 | 0.640 |
| GPT | 1.01 | 1.00-1.01 | 0.027 |  | 1.00 | 0.99-1.01 | 0.764 |
| rGT | 1.00 | 1.00-1.00 | 0.052 |  | 1.00 | 1.00-1.01 | 0.240 |
| ALP | 1.01 | 1.00-1.02 | 0.008 |  | 1.00 | 0.99-1.01 | 0.710 |
| TC | 1.00 | 1.00-1.00 | 0.659 |  | 0.99 | 0.98-1.00 | 0.103 |
| LDL | 1.00 | 1.00-1.00 | 0.827 |  | 1.01 | 1.00-1.02 | 0.231 |
| Uric acid | 1.15 | 1.05-1.26 | 0.003 |  | 1.08 | 0.96-1.22 | 0.209 |
| BUN | 1.04 | 1.01-1.07 | 0.003 |  | 0.99 | 0.95-1.04 | 0.736 |
| Cr | 1.23 | 0.85-1.79 | 0.268 |  | 0.96 | 0.45-2.04 | 0.906 |
| HBV | 0.94 | 0.59-1.50 | 0.81 |  | 1.14 | 0.70-1.87 | 0.600 |
| HCV | 3.11 | 1.86-5.20 | < 0.001 |  | 2.27 | 1.28-4.02 | 0.005 |
| Four phenotypes* | 1.32 | 1.18-1.47 | < 0.001 |  | 1.18 | 1.03-1.34 | 0.015 |

ALP, alkaline phosphatase; ALT, alanine transaminase; AST, aspartate transaminase; Bil-T, total bilirubin; ALP, alkaline phosphatase; AST, aspartate transaminase; Bil-T, total bilirubin; LDL, low-density lipoprotein; TC, total cholesterol; HBV, hepatitis B virus; HCV, hepatitis C virus; *Four phenotypes: MHNO, MHO, MANO and MAO
